# Naringin Chelates Excessive Iron and Prevents the Formation of Amyloid-Beta Plaques in the Hippocampus of Iron-Overloaded Mice

**DOI:** 10.3389/fphar.2021.651156

**Published:** 2021-07-02

**Authors:** Mehrdad Jahanshahi, Masoumeh Khalili, Asra Margedari

**Affiliations:** ^1^ Neuroscience Research Center, Department of Anatomy, Faculty of Medicine, Golestan University of Medical Sciences, Gorgan, Iran; ^2^ Neuroscience Research Center, Golestan University of Medical Sciences, Gorgan, Iran; ^3^ Infectious Diseases Research Center, Golestan University of Medical Sciences, Gorgan, Iran

**Keywords:** iron chelation, brain, hippocampus, naringin, amyloid-β plaque

## Abstract

Metal chelating agents are antioxidant agents, which decrease the reductive potential and stabilize the oxidized metal ion form. In this study, we evaluated the naringin capacity in chelating iron and preventing amyloid-beta plaque formation in the hippocampus of iron-overloaded mice. Thirty-five NMRI male mice (8–10 weeks old) were provided. The mice were classified into five groups. Iron dextran was administered as i.p. injection (100 mg/kg/day) four times a week for four subsequent weeks. The treated groups received 30 and 60 mg/kg/day naringin for a month. After histological processing, the brain sections were stained with Perls’ stain kit for iron spots, and Congo red was used to stain the brain and hippocampus for amyloid-beta plaques. 30 mg/kg/day of naringin was shown to decrease nonheme iron in an efficient manner; iron content in this group decreased to 16.83 ± 0.57 μg/g wet weight, a quantity as low as that observed in the normal saline-receiving group. The nonheme iron content in the mice receiving 60 mg/kg/day of naringin was 20.73 ± 0.65 μg/g wet weight. In addition, Aβ plaque numbers in CA1, CA3, and DG areas of the hippocampus decreased significantly following treatment with 30 or 60 mg/kg/day naringin. Naringin has a strong iron chelation capacity and is able to reduce the formation of amyloid plaques. So it can be useful for neuroprotection and prevention of Alzheimer’s disease.

## Introduction

Alzheimer’s disease (AD) is a common neurodegenerative disorder affecting more than 30 million people around the world that is characterized by amyloid-beta (Aβ) deposition in brain tissues (Chan et al., 2016; Thomas et al., 2020). Aβ chelates heavy metals such as iron, copper, and zinc, resulting in their deposition in the hippocampus, which in turn launches the Fenton reaction (Lovell et al., 1998; Maynard et al., 2002). Hydrogen peroxide species and hydrogen peroxide free radicals (ROS) are the products of the Fenton reaction that cause oxidative damage and lipid peroxidation in brain tissues. Metal chelators chelate iron and formed stable compounds that are excreted through the stool or urine (Liu et al., 2009; Khalili et al., 2015). Metal chelating agents are secondary antioxidants because they reduce the reductive potential and stabilize the oxidized metal ion form. The effective way to decrease Aβ plaques is using a metal chelation agent. Currently used iron chelators, such as deferiprone, deferasirox, and deferoxamine (DFO), are of chemical nature and exhibit diverse side effects (Kontoghiorghes et al., 2010; Grady et al., 2013). So, the search for natural substitutions has been undergone over the last years. Plants have always served as a good source of medication. The flavonoids’ antioxidant activity is increased when they chelate the metal ions (Pereira et al., 2007). Catechin, quercetin, diosmetin, coumarin, anthocyanins, and chalcones are among the herbal compounds that exhibit iron chelation activity (Morel et al., 1993; van Acker et al., 1998a; Fernandez et al., 2002; Macáková et al.,). Baicalin and quercetin have been reported to chelate excessive iron in iron-overloaded mice (Zhang et al., 2006; Zhang et al., 2012). Many compounds are there in nature that alleviates AD symptoms; phenolic compounds found in green tea and curcumin are instances of such compounds. Diverse biological activities have ever been discovered for these compounds, including antioxidant activity, anti-inflammatory activity, iron chelation activity, and neuroprotective effects (Mandel et al., 2007; Choi et al., 2012).

Naringin (the 7-β-neohesperidoside of naringenin [4’,5,7-trihydroxiflavananone]) is a flavanone compound found in abundance in citrus fruits. It has antioxidant activity, metal chelating activity, free radical scavenging properties, antilipid peroxidation, anticancer, and anti-inflammatory activity (Chandra Jagetia et al., 2004; Pereira et al., 2007). Chandra Jagetia et al. (2004) showed that naringin could chelate iron and inhibit the cells from the free radical damage in the *in vitro* condition. Studies show that naringin has been able to cross the brain–blood barrier (BBB) and chelate excessive irons, and it has a neuroprotective effect (van Acker et al., 1998a; Golechha et al., 2011; Alam et al., 2014; Cui et al., 2014). Guo and Sun (2020) reported that naringin could chelate copper (II) and inhibit Cu^2+^-Aβ_1-42_-mediated cytotoxicity on PC12 cells in the *in vitro* condition, and they suggested it is a potential agent for therapy of Alzheimer’s disease. Naringin could improve long-term cognitive function in the transgenic mouse model of AD (Wang et al., 2013).

Therefore, in the current study, we evaluate the naringin capacity in preventing amyloid-beta plaque formation in hippocampus tissues of iron-overloaded mice.

## Materials and Methods

### Metal Chelating Activity Assay

Metal chelation activity of naringin was done according to the methods of Khalili et al. (2015). Briefly, 0.5 ml of 2 mM FeCl_2_ solution was added to 1 ml of naringin (400 μg/ml). Then, 0.2 ml of ferrozine (5 mM) was added, and the solution was kept in the dark place, at room temperature for 15 min. The absorbance of the solution was measured by a spectrophotometer at 562 nm. EDTA was used as a standard (Khalili et al., 2015). The data were calculated using the following equation:

$$I\left(\text{\%}\right)=\left[\frac{\left(A_{\text{Blank}}-A_{\text{Sample}}\right)}{A_{\text{Blank}}}\right]\times 100,$$ (1)

where *A*_Blank_ is the absorbance of the control reaction (containing all reagents except the test sample) and *A*_Sample_ is the absorbance of the fraction.

#### Animal’s Treatment and Experimental Design

Thirty-five NMRI [NMRI (Han) mice strain: NMRI is an outbred mouse strain with white coat color obtained from inbred strain NIH/P1] male mice (8–10 weeks old) were provided. Mice weighed 20–25 g were purchased from Pasteur Institute (Amol, Northern Iran). The mice were kept at the light cycle of 12 h light 12 h dark under controlled temperature (24 ± 2°C) and humidity (45–55%). All the experiments involving the mice were done according to ethical guidelines approved by the Ethical Committee of Golestan University of Medical Sciences, Gorgan, Iran (approval number: ir.goums.rec.1395.274).

The mice were randomly classified into five groups, each consisting of seven mice, and subjected to different treatments after inducing iron-overloaded condition by injection of iron dextran. Iron dextran was administered as intraperitoneal injection (*i.p.*) (100 mg/kg/day) four times a week for four subsequent weeks.

Group 1 (Control) received no iron chelator, Group 2 received iron dextran, Group 3 received deferoxamine (25 mg/kg/day), Group 4 received 30 mg/kg/day naringin, and Group 5 received 60 mg/kg/day naringin. The period between iron dextran injection and the beginning of treatment was one month. Ketamine (90 mg/kg, *i.p.*) and xylazine (10 mg/kg, *i.p.*) were used to insure that the mice were anesthetized for brain excision. Three brain tissues were kept in PBS to quantify their iron contents, and the other brains in each group were maintained in 4% paraformaldehyde solution for Perl’s and Congo red staining.

### Total Nonheme Iron Content in the Brain of the Treated Mice

Brain nonheme iron content was measured according to the method described by Rebouche et al. (2004). Briefly, 100 mg of brain tissue was homogenized in 1 ml high-purity water, using a Dounce homogenizer. 30 µl of the homogenate was added to a microtube containing 1 N HCl and 10% trichloroacetic acid in high-purity water and incubated at 95°C in a bain-marie for 1 h. The microtube was centrifuged at 8,200 g for 10 min; 30 μl of the supernatant was mixed with a mixed solution containing 0.508 mmol/L ferrozine, 1.5 mol/L sodium acetate, and 1.5% (v/v) thioglycolic acid, prepared in high-purity water. After 30 min incubation at room temperature, the absorbance rate was measured at 562 nm using a spectrophotometer (Unico 2,100 Vis spectrophotometer, NJ 08810; United States). The mixture of 30 μl high-purity water and 1.5 mol/L sodium acetate containing 0.1% or 1.5% thioglycolic acid was used as blank. FeSO4 was used as a iron standard (0, 2, 4, 6, 8, and 10 µg/ml), that was diluted with an equal volume of 1 N HCl and 10% trichloroacetic acid in high-purity water. The standard was prepared daily.

### Tissue Preparation

The mice were anesthetized with ketamine and xylazine; their brain tissues were removed and fixed in 4% paraformaldehyde. After a week, the tissues were processed using an automated tissue processing machine (Did Sabz, Urmia, Iran) and embedded in paraffin for histological analysis by Perl’s and Congo red staining method (Nikmahzar et al., 2019). Coronal sections (6 μm) from the hippocampus were taken with a rotary microtome (Pooyan MK 1110, Iran).

### Perl’s Staining

The brain sections were stained with Perls’ stain kit according to the protocol (Shimi Pajhohesh Asia, Amol, Iran). Briefly, after deparaffinization and hydration, the brain sections were placed in potassium ferrocyanide solution and afterward in ferrocyanide-hydrochloric acid solution. The tissues were rinsed with distilled water and counterstained with nuclear fast red solution after another round of rinsing; the tissues were dehydrated in 95% alcohol, absolute alcohol, and cleared in xylene. Finally, the tissues were coverslipped with entellan (Merck, Germany) glue. Pictures were taken using a light microscope (Model: BX 53, Olympus, Japan) at 40× magnification equipped with a digital camera (Model: DP73, Olympus, Japan) (Ebrahimzadeh et al., 2016).

### Congo Red Staining

**FIGURE 1:**
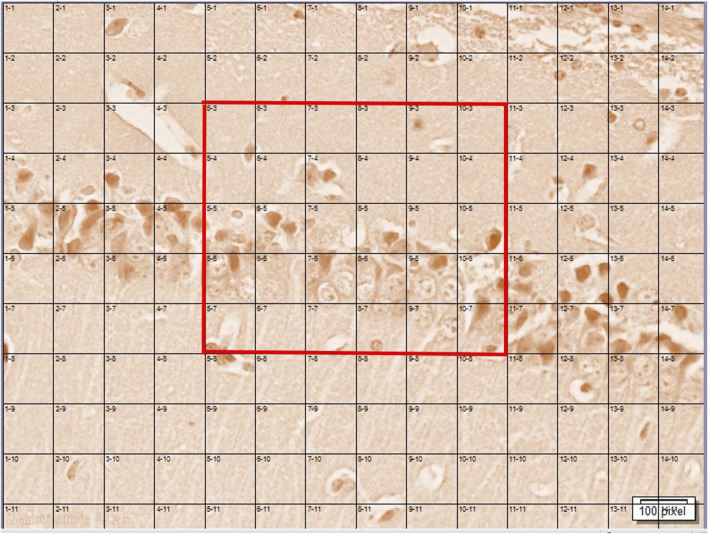
A rectangle consisting of 30 squares (6 × 5) (shown by red color) which were counted for the number of amyloid plaques in the AC1, AC3, and DG areas of the hippocampus.

Congo red staining (DDK, Italia) was used for the detection of congophilic amyloid plaque in the hippocampus. First, the brain sections were deparaffinized and rehydrated by distilled water and then stained with Congo red for 30 min at room temperature. Afterward, the tissues were washed by distilled water and alcoholic solution, rinsed with tap water for 5 min, counterstained with a Mayer hematoxylin solution (for 5 min), and finally washed by running tap water for 10 min. The tissues were rapidly dipped in 95% alcohol (three times) and then immersed twice in 100% alcohol. The tissues were clarified with xylene and coverslipped with entellan glue. In each group, 20 slides of each area (CA1, CA3, and DG area of the hippocampus) were prepared. Pictures of the stained amyloid plaque were taken by using a light microscope (BX53, Olympus, Japan) equipped with a digital camera (DP73, Olympus, Japan) for the hippocampal CA1 and CA3 and dentate gyrus (DG) regions at 40x magnification. Pictures were routed into a Windows PC for quantitative analyses using cellSens Standard 1.14 software (Olympus, Japan). The amyloid plaque was counted in a rectangle consisting of 30 squares (6*5). The means were reported (Figure 1). Imaging and counting were performed blind to the treatment (Jahanshahi et al., 2020).

### Statistical Analysis

Variance analysis (one-way ANOVA) of the data was carried out using GraphPad Prism 5. Means were compared using Newman–Keuls multiple comparison tests. Statistical significance was also set at *p* < 0.05. Means were reported ± SD.

## Results

### Iron Chelation Activity of Naringin in *In Vitro* Condition

The iron chelating activity was measured by the ferrozine/Fe^2+^ methods. Our results show that the iron chelation activity of naringin (concentration 400 μg/ml) was 83 ± 2.01%. EDTA was used as a standard [inhibition concentration 50 (IC50) = 18.27 ± 0.09 μg ml^-1^].

### Total Nonheme Iron in Mice Brain

**FIGURE 2:**
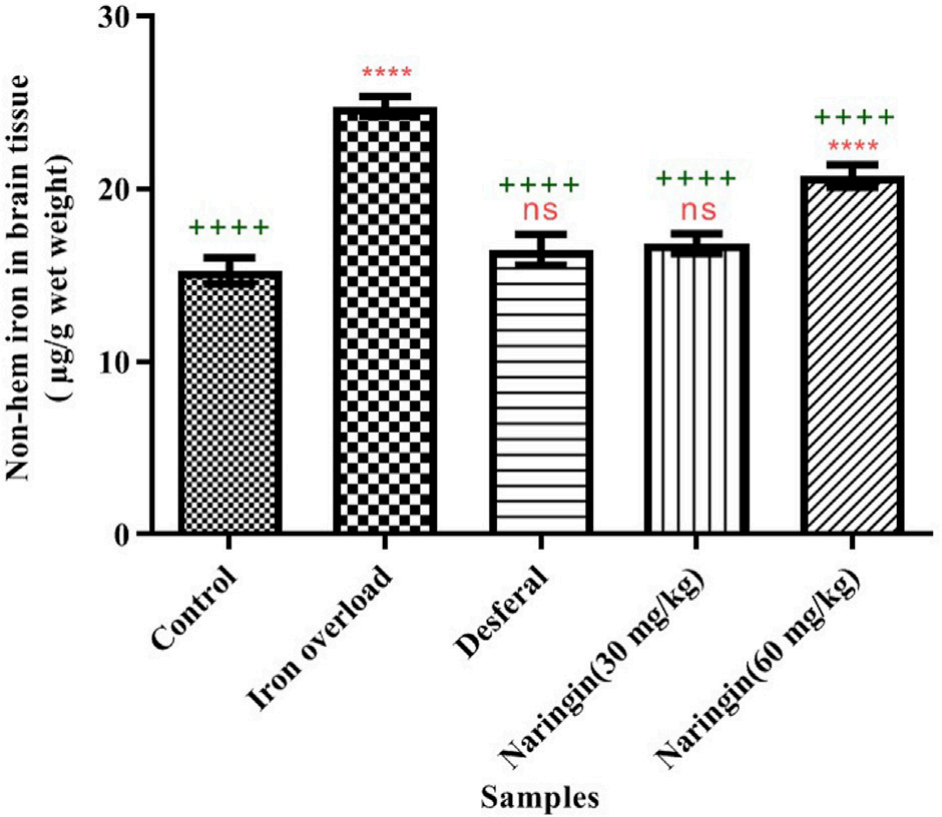
Total nonheme iron content in the brain of different groups. Control group: the mice were treated by normal saline; iron-overloaded group: the mice that were treated by iron dextran; DFO group: iron-overloaded mice that were treated by deferoxamine; naringin 30 mg/kg/day group: iron-overloaded mice that were treated by naringin 30 mg/kg/day; and naringin 60 mg/kg/day group: iron-overloaded mice that were treated by naringin 60 mg/kg/day (means ± SD). “++++” and “ns” indicate probability levels of 0.0001 and nonsignificant, respectively. The stars indicate the probability levels in the same way. “*” shows the comparison of the mean of all groups with the control group. “+” shows the comparison of the mean of all groups with the iron-overloaded group.

Total nonheme iron of the treated and nontreated mice was measured according to the method described by Rebouche et al. (2004). The maximum total iron content (24.77 ± 0.56 μg/g wet weight) belonged to the iron-overloaded group, which is significant compared to the control group (*p* < 0.0001); the minimum quantity (15.27 ± 0.56 μg/g wet weight) belonged to the control group (normal saline-receiving mice). Total nonheme iron content was shown to decrease following treatment with DFO and naringin. As Figure 2 shows, DFO decreases iron content in iron-overloaded mice significantly (16.47 ± 0.88 μg/g wet weight, *p* < 0.001). 30 mg/kg/day of naringin was shown to decrease total nonheme iron in an efficient manner; total iron content in this group decreased to 16.83 ± 0.57 μg/g wet weight, a quantity as low as that observed in the normal saline-receiving group The total nonheme iron content in the mice receiving 60 mg/kg/day of naringin was calculated to be 20.73 ± 0.65 μg/g wet weight.

### Histology: Perl’s Staining

**FIGURE 3:**
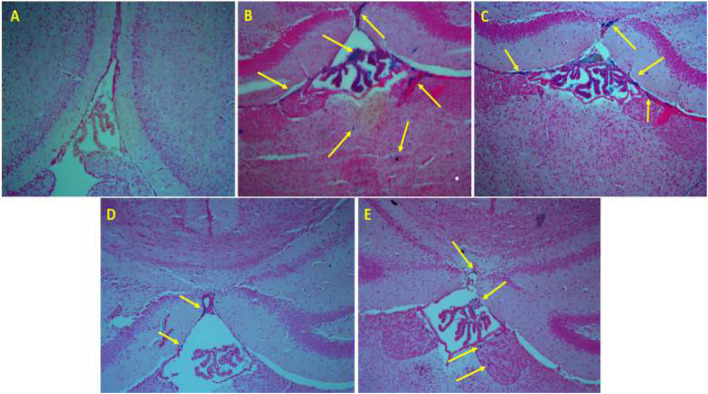
Perl’s staining of the brain tissue. **(A)** The mice that were treated by normal saline, **(B)** the mice that were treated by iron dextran, **(C)** iron-overloaded mice that were treated by DFO, **(D)** iron-overloaded mice that were treated by naringin 30 mg/kg/day, and **(E)** iron-overloaded mice that were treated by naringin 60 mg/kg/day.

The brain’s Perl’s staining results are shown in Figure 3. Blue dots indicate iron deposition (shown by the yellow arrow). Figure 3A shows the tissues of normal saline-receiving mice; no iron deposition is seen. Massive iron deposition occurs in iron-overloaded mice (Figure 3B). Iron deposition was decreased in the iron-overloaded mice following treatment with DFO and naringin (Figures 3C–E).

### Histology: Congo Red Staining

**FIGURE 4:**
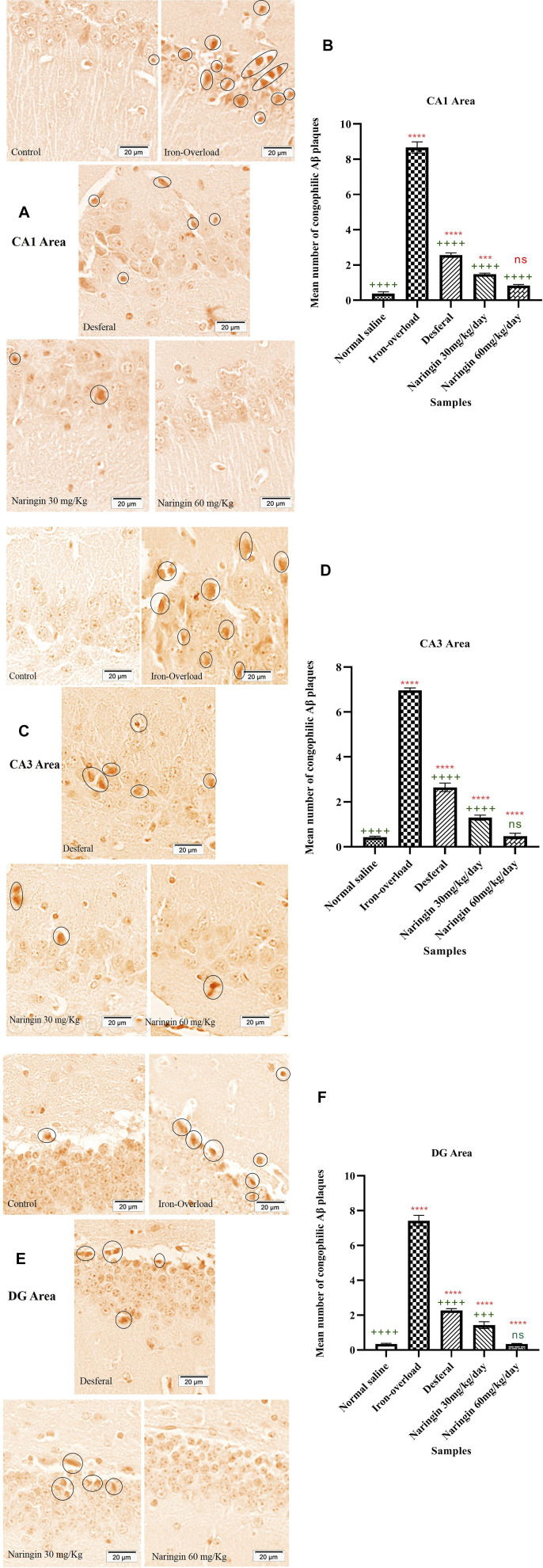
**(A)** and **(B)** CR staining for amyloid-β plaque and the number of amyloid plaques in the hippocampal CA1 area of the mice hippocampus, **(C)** and **(D)** CR staining for amyloid-β plaque and the number of amyloid plaques in the hippocampal CA3 area of the mice hippocampus, and **(E)** and **(F)** CR staining for amyloid-β plaque and the number of amyloid plaques in the hippocampal DG area of the mice hippocampus. Control group: the mice that were treated by normal saline; iron-overload group: the mice that were treated by iron dextran; DFO group: iron-overloaded mice that were treated by DFO; naringin 30 mg/kg/day group: iron-overloaded mice that were treated by naringin 30 mg/kg/day; naringin 60 mg/kg/day group: iron-overloaded mice that were treated by naringin 60 mg/kg/day. Scale bar shows 20 μm. Aβ plaques are red in color, and they are shown by a black circle. “++++,” “+++,” and “ns” indicate probability levels of 0.0001, 0.001, and nonsignificant, respectively. The stars indicate the probability levels in the same way. “*” shows the comparison of the mean of all groups with the control group. “+” shows the comparison of the mean of all groups with the iron-overloaded group.

Congo red staining of the hippocampus and the number of amyloid plaques are shown in Figure 4. Congo red staining of the CA1 areas of the hippocampus shows that the number of Aβ plaques increased up to 8.66 ± 0.32 number/µm^3^ in the iron-overloaded group. After treatment by DFO, the number of Aβ plaques decreased significantly compared with the iron-overloaded group (2.56 ± 0.12 number/µm^3^, *p* < 0.0001). The minimum content of Aβ plaques was obtained in the high dose of naringin (0.83 ± 0.06 number/µm^3^). There was no significant difference between high doses of the naringin group and the normal saline group (Figures 4A,B).

As shown in Figures 4C,D, Aβ plaques in the CA3 area of the hippocampus were affected after the treatment. The maximum content of Aβ plaques was obtained in the iron-overloaded group (6.96 ± 0.10 number/µm^3^); hence, in the control group, Aβ plaques were 0.42 ± 0.04 number/µm^3^. There were significant differences between the iron-overloaded group and the control group (*p* < 0.0001). The content of Aβ plaques dramatically decreased after treatment by DFO and naringin 30 and 60 mg/kg/day (2.64 ± 0.18, 1.29 ± 0.11, and 0.48 ± 0.12 number/µm^3^, respectively). Figures 4E,F show Congo red staining and Aβ plaques content in the DG area of the hippocampus. Aβ plaque content in the DG area of the hippocampus after treatment by iron dextran increased, hence in the iron-overloaded group it increased up to 7.42 ± 0.30 number/µm^3^. The treatment by DFO and naringin caused a decrease in the content of Aβ plaques. The minimum content of Aβ plaques was obtained in the naringin 60 mg/kg/day group (0.34 ± 0.02 number/µm^3^); there were no significant differences between the naringin 60 mg/kg/day group and the control group.

## Discussion

Iron accumulation in the brain tissues causes some neurobrain diseases such as AD and Parkinson’s disease (PD) (Masaldan et al., 2019). Our results show that iron dextran administration leads to iron precipitation in the brain tissues. The excess iron can launch the Fenton reaction and produce hydroxyl radicals. The resultant reactive oxygen species can damage biological molecules and cause different diseases. The fast and effective treatment is using an iron chelator (Fernandez et al., 2002; Mladěnka et al., 2011; Badria et al., 2015).

Studies show that the combination of antioxidant and iron chelator agent may be a significant strategy to decrease symptoms of AD and PD (Mandel et al., 2008). Schrag et al. (2020) show that the brain iron level was increased in PD patients. They revealed that the iron level increased in the hippocampus, thalamus, putamen, and caudate nucleus in PD (Thomas et al., 2020). In this study, we induced iron-overloading condition by injecting iron dextran into mice and found that naringin is able to reduce iron sedimentation rate by chelating excessive iron ions.

**FIGURE 5:**
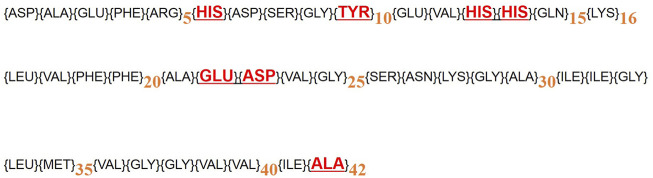
The amino acid sequence of Aβ (1–16), (1–40), and (1–42). Fe (II) and (III) binding sites are shown by red color.

Studies show that Aβ has a high affinity for iron and acts as an iron chelator (Rottkamp et al., 2001). Metals affect Aβ morphology and accelerate the formation of Aβ fibers and increase Aβ toxicity. There is some evidence showing that Aβ binds to Fe (II) and Fe (III). Miura et al. (2001) show that the N-terminal hydrophilic segment of Aβ binds to Fe (III). The N-terminal of a hydrophilic segment of Aβ1-16 has three histidines (His6, 13, and 14) and one tyrosine (Tyr10), which binds to Fe (II) and (III) (Miura et al., 2001). Also, Fe^3+^ ions bind to Aβ1–40, which has residues at positions 17–40, in individual Glu22 and Asp23, that may provide binding sites for Fe(III) (Miura, Suzuki and Takeuchi 2001). About Aβ 1–42, Fe^3+^ binds between Glu22 and the carboxyl group of the Ala42 at C-terminus. Fe2^+^ and Fe^3+^ cause aggregation of Aβs (Azimi and Rauk 2012; Roy et al., 2020; Ryu et al., 2008; Vahed et al., 2019) (Figure 5). An increase in the iron level led to an increase in both Aβ oligomers, and Congo red was used to stain the brain for amyloid-beta plaques (House et al., 2004; Guo et al., 2013; Becerril-Ortega et al., 2014). The combination of iron with Aβ produces H_2_O_2_, which may produce hydroxyl free radicals during the Fenton reaction, resulting in cellular oxidative damage. Thus, natural compounds can be useful in reducing the symptoms of AD by chelating metals from Aβ fibers and inhibiting free radicals (Bandyopadhyay and Rogers, 2014). Iron chelators are one of the main treatments for AD due to iron deficiency in the brain and the formation of protein plaques from amyloid precursors (Ruch et al., 1989). Our results showed that naringin reduced the levels of Aβ plaques in the hippocampus of the mice brain. Investigation of the amyloid plaques in the hippocampus showed that naringin is able to reduce the number of amyloid plaques in mice. Iron can accumulate in the cerebellum, hippocampus, nucleus accumbens, and substantia nigra (Masaldan et al., 2019). Naringin reduced the iron content in mice brains. Various studies have shown that natural compounds reduce oxidative stress in the brain tissue and decrease the rate of reactive oxygen species production. Resveratrol reduced oxidative stress in the brains of treated rats (Mokni et al., 2007).

Neuronal protection and iron chelation activity have been reported for other natural compounds as well. For instance, Mandel et al. (2008) reported that catechin of green tea reduces the adverse effects of AD and PD. Iron chelation capacity has been reported for several natural compounds, among which are curcumin, baicalin, apocynin, and quercetin (Davinelli et al., 2013; Chan et al., 2016; Gu et al., 2016; de Andrade Teles et al., 2018).

Iron is an important factor for neurons to act normally; however, its accumulation in the brain causes several disorders. Thomas et al. (2020) show that brain iron content measures can be used to probe key clinical indices of PD activity. Iron chelation therapy is a new strategy for treating diseases like AD and PD. Early detection of iron accumulation in the brain and the use of iron chelators may prevent iron-related brain diseases (Zecca et al., 2004).

## Conclusion

Naringin is a flavanone compound found in abundance in citrus fruits and has a strong iron chelation capacity. Naringin could reduce the formation of amyloid plaques in the hippocampus and chelate excessive iron from the iron-overloaded mice’s brain. Therefore, it can be useful for the protection of neurons in some neurodegenerative diseases related to iron.

## Data Availability

The raw data supporting the conclusions of this article will be made available by the authors, without undue reservation.

## Abbreviation

AD: Alzheimer’s disease; PD: Parkinson’s disease; Aβ: amyloid-beta; ROS: hydrogen peroxide free radicals; BBB: brain–blood barrier; i.p.: intraperitoneal injection; DFO: deferoxamine.
